# Prostate volume: does it predict patient outcomes following prostate artery embolisation? A retrospective cohort study

**DOI:** 10.1186/s42155-024-00464-4

**Published:** 2024-06-27

**Authors:** Robert Wise, Howell Fu, Charles Ross Tapping

**Affiliations:** Department of Radiology, Oxford University Hospitals NHS Foundation Trust John Radcliffe Hospital, Headley Way, Headington, Oxford, OX3 9DU United Kingdom

**Keywords:** Prostate artery embolisation, Prostate size, Prostate volume, Lower urinary tract symptoms

## Abstract

Prostate artery embolisation (PAE) is a minimally invasive procedure commonly performed to treat lower urinary tract symptoms (LUTS) secondary to benign prostatic hyperplasia. International Prostate Symptom Score (IPSS) is a validated patient questionnaire quantifying LUTS and is used for patient selection for PAE, but it is largely subjective. Prostate volume is an easily estimated objective parameter across multiple imaging modalities. No strict threshold of prostate volume is established as a selection criterion for PAE, but it is generally accepted that prostate volume should be over 40 to 50 mL.

We looked at a sample of 65 cases performed at a large teaching hospital between 2017 and 2019 with a minimum of four years follow up. Embospheres between 100 to 500 microns were injected into the prostatic arteries bilaterally (if technically feasible). A ‘bullet shape’ model was used to estimate prostatic volume from initial CT. *N* = 13 had an estimated volume < 51 mL (range 31-50 mL). IPSS before and at 3 months post-procedure were collected.

80% of patients indicated a beneficial response to PAE (IPSS improvement > 5). 23% of patients required further PAE procedure or surgery. No major complications were recorded. The mean change in IPSS under 51 mL compared to over 51 mL cohort was 10.2 versus 11 (standard deviation 7.5 versus 7.3) (*p* = 0.44, 2 tailed Student’s T-test).

There was no statistically significant difference in the IPSS improvement or outcome of small volume prostates under 51 mL compared to large volume. Our results suggest that prostate volume should not be used to exclude patients for PAE.

## Introduction

Prostate artery embolisation (PAE) was first described in 2000 [1] but has recently gained in popularity as a minimally invasive alternative for managing lower urinary tract symptoms (LUTS) associated with benign prostatic hyperplasia (BPH). BPH is characterised by non-malignant enlargement of the prostate gland. This enlargement can impinge on the urethra, leading to a spectrum of LUTS that significantly affect the quality of life, including increased frequency, urgency, a weakened stream, and nocturia. LUTS are widespread, and becomes more prevalent with age, affecting up to 90% of men above 80 years [2].

Traditionally, treatment options have ranged from medication to various forms of surgery, each with its own set of risks and benefits [3]. PAE offers a novel approach by targeting the arteries supplying the prostate to reduce its size and alleviate symptoms. The procedure involves the catheter-based introduction of embolic materials to obstruct the prostatic arteries, thereby reducing blood flow and causing the prostate to shrink [2].

The attractiveness of PAE lies in the elimination of hospital stays, reduction in typical surgical risks, and a lower likelihood of sexual health complications such as retrograde ejaculation or erectile dysfunction [4, 5]. Additionally, PAE is often performed under local anaesthesia, reducing the risks associated with general anaesthesia [5, 6].

The International Prostate Symptom Score (IPSS) is an essential tool in the clinical assessment of patients with lower urinary tract symptoms (LUTS) due to benign prostatic hyperplasia (BPH). As a validated questionnaire, the IPSS allows patients to self-report the severity of their symptoms, providing a subjective measure that helps clinicians in both diagnosing BPH and determining its impact on the patient's quality of life [7]. While the IPSS is useful for patient selection for PAE, it is intrinsically subjective and does not incorporate volume measurements.

Prostate volume can be consistently measured across various imaging modalities, including ultrasound, MRI, and CT scans. Despite its ease of estimation and its potential importance in treatment planning, there is currently no strict criterion regarding prostate volume for selecting patients for PAE in published guidelines [4].

The Cardiovascular and Interventional Radiology Society (CIRSE) Standards of Practice on PAE inclusion criteria suggests offering treatment for a prostate volume > 30–50 mL [2]. The Society of Interventional Radiology Multisociety Consensus Position Paper does not offer a recommendation on prostate size [8]. The American Urological Association BPH guidelines suggest further randomised control trials are required on PAE as a treatment and do not recommend its routine clinical use [9].

The primary literature presents mixed evidence regarding the role of prostate size as a discriminator for PAE outcomes, with some studies showing that larger prostates benefit more from PAE but others finding no such relationship. Despite these varied findings, there seems to be a general consensus in published literature that a prostate volume of over 40 to 50 ml might be considered a reasonable threshold for PAE candidacy. This threshold is based on the notion that larger prostates are more likely to benefit from the volume reduction achieved through arterial embolization [2, 4, 10].

The aim of this work is to assess the subjective IPSS benefit of PAE for patients with smaller prostates (under 50 mL) compared to larger prostates (over 50 mL).

## Materials & methods

A retrospective review was performed of all consecutive cases performed at a large teaching hospital between 2017 and 2019. This study period was chosen to ensure adequate follow-up period of over 4 years, while maximising sample size. 5 cases were excluded due to absence of IPSS data, leaving 65 cases included in the dataset. The indication for all of the procedures was lower urinary tract symptoms. Initial prostate volume was estimated using CT, which was already available due to all patients having planning CTs to assess the anatomy for embolisation. A "bullet shape" model (volume = length x height x width x [pi/4.8]) was used to estimate prostatic volume from dimensions measured on initial planning CT by the study’s author. This has been shown to provide a better representation of prostate volume for small prostate glands [11].

Embolisations were performed by multiple operators, including subspecialty interventional radiology trainees, and always including a consultant for every procedure. All patients underwent pump injected cone beam CT. The prostatic arteries were embolised with Tris-acryl gelatin microspheres ("Embospheres, Merit Medical") bilaterally (if technically feasible) until contrast stasis was achieved. Bilateral embolisation was achieved in 90.7% (59) cases. Reasons for technical failure were related to target vessel stenosis, or unable to locate the target vessel on imaging. 33 patients received 200 μm Embospheres, 31 patients received 300–500 μm Embospheres, and one patient received 100 μm Embospheres. The variation in particle size was due to individual radiologists's preferences, which differed because many of the early cases were performed before firm evidence for optimal particle sizes had been established in the literature.

Patients were followed up with regular clinic review and IPSS scores, and cases requiring further intervention or surgery were noted. All patients filled in and returned paper questionnaires indicating their IPSS shortly before the procedure and at three months post-procedure. An improvement of 5 IPSS points or above was considered significant clinical response. Some previous authors have suggested a difference of less than 3 IPSS points as significant clinical improvement [12], but our institutional experience suggests that a 5 point threshold should provide more clinically robust results.

## Results

Average case follow-up period was 68.8 months, (range 38 to 99). 13 cases had an estimated volume < 51 mL (range 31- 50 mL, mean 41, “Small prostates”), 52 cases had an estimated volume > 51 mL (range 55 – 239 mL, mean 115, “Large prostates”). Bilateral embolisation was achieved in 90.7% (59) cases. Unilateral embolisation was performed only if one side was inaccessible: this was due to vessel stenosis in 3 patients and being unable to identify the prostatic artery origin in 3 patients. Approximately equal numbers of patients received small vs large Embospheres, in both the small and large prostate volume groups (Table 1).

**Table 1:** Size of embolic particles delivered by prostate volume cohort

	100 μm Embospheres (n)	200 μm Embospheres (n)	300–500 μm Embospheres (n)
Small volume cohort	0	6	7
Large volume cohort	1	27	24

21.5% of cases [14] required a repeat treatment or further intervention including transurethral resection of the prostate (TURP), due to persistent or recurrent LUTS. Of the cohort receiving 200 μm Embospheres, 33% [11] required further intervention. Of the cohort receiving 300–500 μm Embospheres, 9.6% [3] required further intervention. The cases receiving 200 μm Embospheres were performed at the beginning of the study period, and therefore had the longest follow-up period and were early in the learning curve of consultant operator experience, both of which may account for the difference in rate of further intervention. None of the 5 unilaterally treated patients required further intervention. No major complications were recorded.

**Fig. 1:**
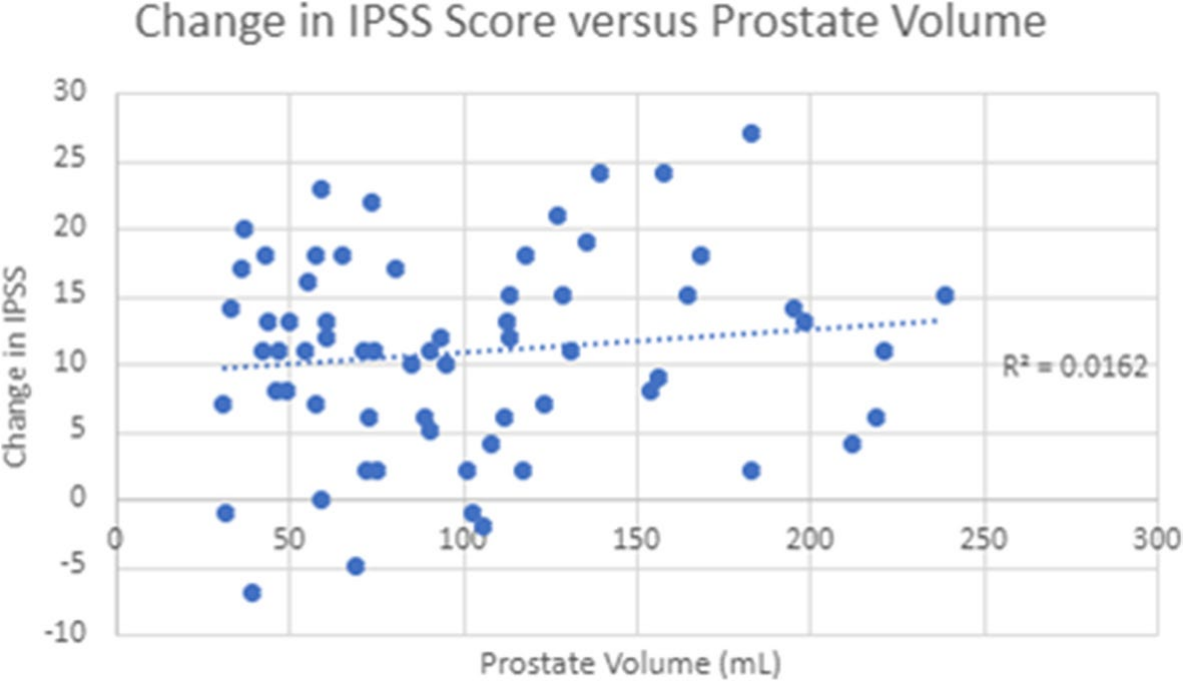
Scatter plot of change in IPSS vs. estimated initial prostatic volume

IPSS average pre-procedure was 21.7 (range 12–36, s.d. 6.2). At 3 months follow up average IPSS was 10.9 (range 1–26, standard deviation 6.3). The mean change in IPSS in the small prostates cohort compared to the large prostates cohort was 10.2 vs 11.0 (s.d. 7.5 vs 7.3) (see Table 2). Figure 1 plots change in IPSS versus initial prostate size, and demonstrates no association using correlation coefficient (*r* = 0.0162). This was not statistically different between the two cohorts (*p* = 0.44, 2 tailed Student’s T-test). The rate of reintervention was 15% vs 23% in the small and large prostates cohorts respectively.

**Table 2:** Cohort demographics, prostatic volume, follow up period and outcome

	**n**	**Mean Age (years)**	**Smoking history positive (%)**	**Hypertension history (%)**	**Mean Prostatic Volume (mL)**	**Baseline IPSS**	**Follow up IPSS**	**Change in IPSS**	**Mean follow up (months)**	**Reintervention rate within 4 years**
**Small volume cohort**	13	69.8 (SD 8.8)	15.3	31	40.9 (SD 6.5)	23.5	13.4	10.2 (SD7.5)	64.5 (SD 22.2)	2 (15%)
**Large volume cohort**	52	69.8 (SD 8.0)	17.3	38.5	115.7 (SD 50.1)	21.2	10.3	11.0 (SD 7.3)	69.8 (SD 18.2)	12 (23%)

## Discussion

Our results suggest that smaller prostates do not necessarily have poorer outcomes after PAE. It is natural to assume that larger prostates cause greater mechanical obstruction of the urinary tract, and may therefore stand to benefit more from PAE. However, in our cohort, there was no significant difference in the level of subjective symptomatic improvement between small volume (less than 51 mL) and large volume prostates, as measured by the IPSS. This supports the view that the determinants of PAE outcomes are complex, and that baseline prostate volume, while easy to measure, has poor predictive power in determining which patients are most likely to benefit. Furthermore, in our data, the small volume prostate cohort experienced a lower rate of reintervention, which may add to patient satisfaction in the long term. Previous authors have reported up to 60.4% recurrence rate and 50.8% reintervention rate occurred within 5 years [13].

Our data also suggests the rate of recurrence is higher in patients receiving smaller particle sizes. However, it should be noted that these patients receiving 200 μm Embospheres were treated earlier in the program and therefore have received a longer follow-up period. Furthermore, operator experience and familiarity with the use of cone-beam computed tomography (CBCT) have increased over the study period, which would also tend to reduce recurrence.

Upon review of the existing literature, the largest studies establishing the safety and efficacy of PAE did not analyse outcomes by baseline prostate volume, although they included prostates as small as 20 ml [5, 14]. Some regression modelling has linked larger baseline prostate volume with more favourable symptomatic relief. For example, in a prospective cohort of 86 patients, regression modelling showed greater IPSS improvements in larger prostates (coefficient = 0.2, *p* = 0.049) [15]. A more recent study of 125 patients found no effect of baseline prostate volume on the recurrence rate of LUTS in regression modelling, although unilateral PAE was a significant predictor of recurrence (*p* < 0.05) [13]. The baseline prostate volume in these studies were generally large (respectively mean 91.8 ml and median 98.43 ml), with very few < 50 ml.

To our knowledge, only one study has directly compared outcomes in prostates above and below 50 ml. A 2015 study involving 78 patients found no significant difference in outcomes between smaller (< 50 ml), medium (50–80 mL) and large (> 80 mL) prostates as measured by the American Urological Association (AUA) symptom index [16]. This suggests that prostate size alone may not be a reliable selector of PAE success.

Another study from 2015 [17] involving 115 patients indicated better outcomes in larger than medium-sized prostates (> 50 ml vs. > 80 ml) when treated with 100 μm embolic particles. However, no prostates below 50 ml were included in the cohort.

Limitations of our study include the sample size (*n* = 65) and the variation in the size of embolic particles used. Bearing these in mind, our findings appear to align with other studies questioning the use of prostate volume as a patient selector for PAE. Instead, alternative technical aspects could potentially offer more reliable indicators for predicting successful outcomes. For example, severe vascular calcification, small vessel size, and significant vessel tortuosity are known to increase the technical difficulty of the procedure, have been associated with longer procedure time and unilateral embolisation, and appear in guidelines as relative contra-indications to PAE [2, 15, 18–20].

Future research in this area may be able to link these technical factors directly to patient-centered outcomes such as IPSS. Emerging imaging technologies like photon-counting CT (PCCT) and calcium subtraction are reducing artefact and improving the diagnosis of coronary calcification in cardiac imaging [21], and leveraging them in prostate imaging may provide more robust predictors of treatment success.

Nevertheless, it is crucial to note that despite the evolving understanding of patient selection criteria, PAE remains a relatively safe and effective treatment within a spectrum of options. As we continue to expand our understanding of the procedure's application in managing lower urinary tract symptoms, it is likely that more sophisticated methods will develop to ensure that the patients most likely to benefit can be identified. In addition to clinical assessment and operator experience, this may involve a combination scoring of specific characteristics based on planning imaging. Although easy to determine, prostate volume is likely to be of little value in predicating final patient satisfaction.

## Conclusion

Our data demonstrated no statistically significant difference in IPSS improvement of small volume prostates (< 51 mL) compared to large volume. These results suggest that small prostate volume should not be considered a contra-indication to PAE, and support the ongoing absence of a prostate volume criterion from best practice guidelines. Further research may be able to define other clinical, imaging, and angiographic variables which better predict IPSS improvement.

## Data Availability

The datasets used and/or analysed during the current study are available from the corresponding author on reasonable request.
